# Decoding the role of angiopoietin-like protein 4/8 complex–mediated plasmin generation in the regulation of LPL activity

**DOI:** 10.1016/j.jlr.2023.100441

**Published:** 2023-09-04

**Authors:** Yan Q. Chen, Eugene Y. Zhen, Anna M. Russell, Mariam Ehsani, Robert W. Siegel, Yuewei Qian, Robert J. Konrad

**Affiliations:** Lilly Research Laboratories, Eli Lilly, and Company, Indianapolis, IN, USA

**Keywords:** angiopoietin-like protein (ANGPTL), apolipoprotein (Apo), lipoprotein lipase (LPL), plasmin, plasminogen, tissue plasminogen activator (tPA), triglycerides (TG)

## Abstract

After feeding, adipose tissue lipoprotein lipase (LPL) activity should be maximized, therefore the potent LPL-inhibitory activity of angiopoietin-like protein 4 (ANGPTL4) must be blocked by ANGPTL8 through formation of ANGPTL4/8 complexes. ANGPTL4/8 tightly binds and protects LPL but also partially inhibits its activity. Recently, we demonstrated ANGPTL4/8 also binds tissue plasminogen activator (tPA) and plasminogen to generate plasmin that cleaves ANGPTL4/8 to restore LPL activity. Although fully active LPL in the fat postprandially is desirable, ANGPTL4/8 removal could subject LPL to profound inhibition by ANGPTL3/8 (the most potent circulating LPL inhibitor), inhibition by other LPL inhibitors like ANGPTL4, ANGPTL3, and ApoC3 or interfere with ApoC2-mediated LPL activation. To understand better these potential paradoxes, we examined LPL inhibition by ANGPTL3/8, ANGPTL4, ANGPTL3, and ApoC3 and LPL stimulation by ApoC2 in the presence of ANGPTL4/8 + tPA + plasminogen. Remarkably, ANGPTL3/8-mediated LPL inhibition was almost completely blocked, with the mechanism being cleavage of fibrinogen-like domain–containing ANGPTL3 present in the ANGPTL3/8 complex. The LPL-inhibitory effects of ANGPTL4, ANGPTL3, and ApoC3 were also largely reduced in the presence of ANGPTL4/8 + tPA + plasminogen. In contrast, the ability of ApoC2 to stimulate LPL activity was unaffected by ANGPTL4/8-mediated plasmin generation. Together, these results explain how plasmin generated by increased postprandial ANGPTL4/8 levels in adipose tissue enables maximal LPL activity by preventing ANGPTL3/8, ANGPTL4, ANGPTL3, and ApoC3 from inhibiting LPL, while permitting ApoC2-mediated LPL activation to occur.

After feeding, lipoprotein lipase (LPL) in adipose tissue should be maximally active on capillary luminal surfaces so that circulating triglycerides (TG) can be hydrolyzed to yield FAs that can then be taken up into adipocytes for re-esterification and subsequent storage as intracellular TG (1, 2). For this to happen, LPL secreted by adipocytes into the subendothelial space must be translocated across the capillary endothelium by glycosylphosphatidylinositol high-density lipoprotein–binding protein 1 (3, 4, 5). While this occurs, LPL must be shielded from inactivation by localized N-terminally intact angiopoietin-like protein 4 (ANGPTL4), a potent inhibitor of LPL that is also secreted by adipocytes into the subendothelial space (6, 7, 8).

Intact ANGPTL4 works mainly in an autocrine/paracrine manner, as its circulating levels are much lower than those of other ANGPTL proteins and complexes (9). Protection from the potent LPL-inhibitory effect of localized ANGPTL4 occurs through increased levels of ANGPTL8, leading to the formation of increased amounts of ANGPTL4/8 complexes, which reduce the ability of ANGPTL4 to inhibit LPL (9, 10). The ANGPTL4/8 complex tightly binds and protects LPL from ANGPTL4 inhibition but also moderately inhibits LPL’s enzymatic activity (11). Recently, we showed that ANGPTL4/8 also binds tissue plasminogen activator (tPA) and plasminogen, to catalyze the generation of plasmin, which can then cleave ANGPTL4/8 to restore LPL activity (11).

This observation suggested a possible conundrum, however. Although fully active LPL in the fat would be desirable after feeding, plasmin-mediated cleavage of ANGPTL4/8 on luminal capillary surfaces could also leave LPL in an unprotected state, susceptible to inhibition by ANGPTL3/8, the most potent circulating LPL inhibitor and whose levels increase dramatically after feeding (9, 10, 11, 12, 13). In addition, unprotected LPL could also be vulnerable to inhibition by the other major circulating LPL inhibitors ANGPTL3 and ApoC3, as well as by any localized ANGPTL4 that might be present (14, 15, 16, 17, 18, 19, 20, 21, 22). Although ANGPTL3 and ApoC3 are much less potent LPL inhibitors than the ANGPTL3/8 complex and their concentrations do not change dramatically when transitioning from the fasted to the fed state (9), they are still capable of inhibiting LPL on luminal capillary surfaces in adipose tissue. Likewise, any ANGPTL4 present in the local environment would also be capable of inhibiting LPL activity. Furthermore, plasmin generation in adipose tissue capillaries to cleave the ANGPTL4/8 complex and remove it from LPL might also possibly impair ApoC2-mediated LPL activation. ApoC2 is well known to be a critical LPL activator required for TG hydrolysis, as evidenced by the fact that ApoC2 deficiency causes severe hypertriglyceridemia (23, 24, 25, 26, 27).

To understand better each of these potential paradoxes, we examined the respective abilities of ANGPTL3/8, ANGPTL3, ANGPTL4, and ApoC3 to inhibit LPL activity and the ability of ApoC2 to stimulate LPL activity after incubation of LPL with ANGPTL4/8 in the presence of tPA + plasminogen. In our current study, we show that ANGPTL4/8-mediated plasmin generation largely blocks the ability of ANGPTL3/8, ANGPTL3, ANGPTL4, and ApoC3 to inhibit LPL activity, while still allowing ApoC2 to be able to stimulate LPL activity. Together, these data provide a plausible explanation for how ANGPTL4/8-mediated plasmin generation in adipose tissue enables LPL to be maximally active in the fat in the postprandial state.

## Materials and Methods

### Recombinant proteins and antibodies

Recombinant human tPA (ab92637) was purchased from Abcam. Human plasminogen (1939-SE), anti-ApoC2 rabbit antibody (MAF4497), anti-ANGPTL3 rabbit polyclonal antibody (AF3829), and anti-LPL antibody (AF7197) were purchased from R&D systems. An anti-His antibody was generated internally using methods previously described (9). Anti-ANGPTL3 and anti-His antibodies were biotinylated using EZ-link sulfo-NHS-LC-biotin (A39257) (Thermo Fisher Scientific). Alexa 680–conjugated anti-rabbit antibody (AF32734), Alexa 680–conjugated streptavidin (S21378), and Alexa 680–conjugated anti-goat antibody (A32860) were obtained from Thermo Fisher Scientific. Human ApoC2 (NBP1-99292) was purchased from Novus Biologicals (Centennial, CO). Recombinant human ANGPTL3, ANGPTL4, ANGPTL4/8, and ANGPTL3/8 were expressed as previously described (9, 11). C-terminal His-tagged human ApoC3 (NP_000031.1) was produced transiently in HEK293 cells and purified through nickel-nitrilotriacetic acid affinity, followed by size-exclusion chromatography.

### Effects of ApoC3 and ApoC2 on LPL enzymatic activity

To study the effects of ApoC3 and ApoC2 on LPL enzymatic activity, human HEK293 LPL stable expressing cells were incubated with increasing amounts of each protein. Incubations were carried out at 37°C using assay medium (DMEM/F12 medium containing 0.1% FA-free BSA). After overnight incubation of the LPL stable expression cells in growth medium, the medium was replaced with 80 μl of assay medium containing increasing concentrations of ApoC3 or ApoC2 protein, and cells were incubated for 60 min. Afterward, 20 μl of 5× working solution, freshly prepared with 0.05% Zwittergent detergent 3-(N,N-dimethyl-octadecylammonio)-propanesulfonate (Sigma) containing EnzChek lipase substrate BODIPY dabcyl–labeled TG analog (Invitrogen), were added to achieve a final substrate concentration of 1 μM. At the end of each experiment, fluorescence was monitored with a Synergy Neo2 plate reader at 1 and 30 min with an excitation wavelength of 485 nm and an emission wavelength of 516 nm.

### Human LPL activity assays with ANGPTL proteins and apolipoproteins

LPL activity assays were performed in LPL stable–expressing HEK293 cells as previously described (11) with minor modifications. All incubations were carried out at 37°C using assay medium (DMEM/F12 medium containing 0.1% FA-free BSA). In each experiment where tPA and plasminogen were added, the final concentration of tPA was 0.1 nM and the final concentration of plasminogen was 30 nM. In experiments where ANGPTL3/8, ANGPTL4, ANGPTL3, or ApoC3 were added for a penultimate incubation prior to the addition of lipase substrate, final concentrations of 0.6 nM, 10 nM, 10 nM, or 0.5 μM were added, respectively, with the concentrations corresponding to the approximate IC_70-90_ for ANGPTL3/8, ANGPTL4, and ANGPTL3 (9) and to the approximate IC_50-60_ of the much less potent LPL inhibitor ApoC3. In experiments when ApoC2 was added for a penultimate incubation prior to the addition of lipase substrate, a final concentration of 50 nM ApoC2 was used based on our previous work (27) as well as the ApoC2 stimulation of LPL activity observed in our current study. After overnight incubation of human LPL stable expression cells in growth medium, the medium was replaced with 60 μl of assay medium, and cells were incubated for 60 min in the absence or presence of 100 nM ANGPTL4/8.

Afterward, 10 μl of assay medium containing either vehicle alone or tPA + plasminogen were added to the cells, and the incubation was continued for 30 min. Next, 10 μl of assay medium containing either vehicle alone, ANGPTL3/8, ANGPTL3, ANGPTL4, ApoC3, or ApoC2 were added, and the incubation was continued for a further 90 min. Finally, 20 μl of 5× working solution, freshly prepared with 0.05% Zwittergent detergent 3-(N,N-dimethyl-octadecylammonio)-propanesulfonate (Sigma) containing EnzChek lipase substrate BODIPY dabcyl–labeled TG analog (Invitrogen), were added to achieve a final substrate concentration of 1 μM for a further incubation of 30 min. At the end of each experiment, one part of each sample had its fluorescence monitored with a Synergy Neo2 plate reader with an excitation wavelength of 485 nm and emission wavelength of 516 nm. In another part, Western blotting was performed as described below.

### Western blotting experiments

At the end of the final incubation period for each LPL activity assay, the reaction was stopped in a portion of the sample by the addition of an equal volume of SDS-PAGE sample buffer, and samples were boiled for 5 min. Samples were separated on gels and transferred to separate PVDF membranes. The membranes from cells treated with ANGPTL3/8 and ANGPTL3 were probed with biotinylated anti-ANGPTL3 rabbit polyclonal antibody, and detection was performed with Alex-680–conjugated streptavidin. Membranes from cells treated with ApoC3 were probed with a biotinylated anti-His antibody, and detection was performed with Alex-680–conjugated streptavidin. Membranes from cells treated with ApoC2 were probed with rabbit anti-ApoC2 antibody, and detection was performed with Alex-680–conjugated anti-rabbit antibody. All images were recorded using a LI-COR Odyssey imaging system. For the membranes from the cells treated with ANGPTL3, quantification of the intensity of the ANGPTL3 bands was performed using LI-COR software.

### Plasmin activity assays

The ability of tPA to convert plasminogen to plasmin in the presence of ANGPTL4/8, ANGPTL4/8 + ANGPTL3/8, ANGPTL4/8 + ANGPTL3, ANGPTL4/8 + ANGPTL4, ANGPTL4/8 + ApoC3, and ANGPTL4/8 + ApoC2 was determined using a colorimetric plasmin activity assay as previously described (11) with minor modifications. Briefly, in these experiments, the concentration of tPA was set at 0.2 nM, the concentration of ANGPTL4/8 was set at 200 nM, and each additional protein was added at concentrations to achieve the exact same molar ratios to the ANGPTL4/8 complex that were used in the LPL enzymatic activity assays. Samples were incubated at 37°C in assay buffer (Abnova) containing varying amounts of plasminogen (0–1200 nM) and 1 mM of the colorimetric plasmin substrate D-Val-Leu-Lys-pNA, and absorbance at 405 nm was recorded using a Molecular Device spectrometer.

### Characterization of LPL following ANGPTL4/8-mediated plasmin generation

To characterize the effects of ANGPTL4/8-mediated plasmin generation on LPL, HEK293 LPL stable–expressing cells were first incubated with 100 nM ANGPTL4/8 for 1 h at 37°C in assay medium. Afterward, cells were incubated in the absence or presence of tPA (0.1 nM) + plasminogen (30 nM) for 2 h at 37°C. Cells were washed, fresh media supplemented with 0.1 U/ml heparin was added to detach membrane-bound LPL, and the incubation was continued at 37°C for 1 h. The amount of membrane-bound LPL in each sample was analyzed via Western blotting as previously described (11). Cells remaining in the wells were solubilized using RIPA buffer containing protease inhibitors and DNAase, and LPL present within the cells was measured via Western blotting as previously described (11). For each LPL analysis, PVDF membranes were probed with goat anti-LPL antibody, followed by Alexa 680–labeled anti-goat antibody.

### Data analysis

In all LPL activity assays, the fluorescent signal was recorded at 1 and 30 min, and the 1 min reading was subtracted from the 30 min reading to correct for background. In each experiment, the vehicle control fluorescent signal corresponded to 100% LPL activity, and the amount of LPL activity present in all other samples was expressed as a percent of the vehicle control. In each LPL activity assessment, significance for the effect of the combination of proteins of interest on LPL enzymatic activity was calculated using an unpaired *t* test to compare the respective treatment group to the most relevant condition. A *P*-value of <0.05 was considered to indicate statistical significance.

## Results

### ANGPTL4/8-mediated plasmin generation blocks the ability of ANGPTL3/8 to inhibit LPL

**Fig. 1:**
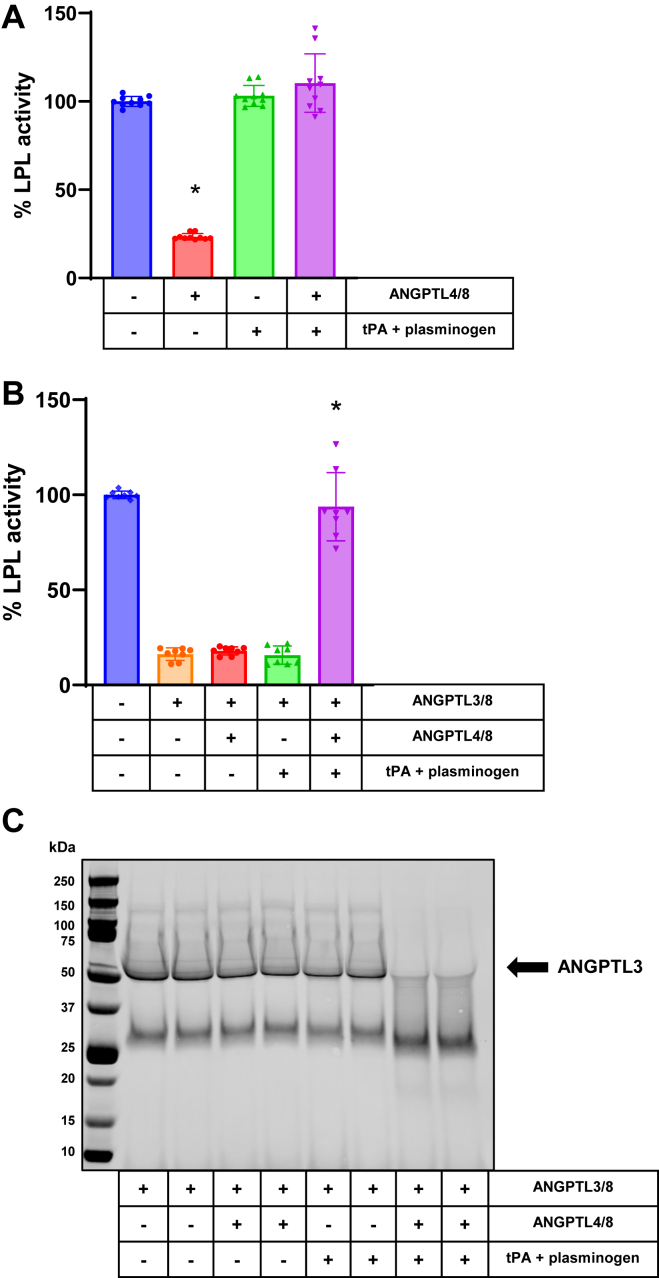
ANGPTL4/8-mediated plasmin generation blocks ANGPTL3/8-mediated LPL inhibition. A: LPL stable expression cells were incubated with lipase substrate after being previously incubated with vehicle alone, ANGPTL4/8, tPA + plasminogen, or ANGPTL4/8 + tPA + plasminogen. LPL activity was calculated as a percent of vehicle control. Results are shown as the mean ± SD (n = 10 from 4 independent experiments) (∗*P* < 0.001 vs. vehicle). B: LPL stable expression cells were incubated with vehicle alone throughout the experiment or in the absence or presence of ANGPTL3/8 after being previously incubated with vehicle alone, ANGPTL4/8 alone, tPA + plasminogen, or ANGPTL4/8 + tPA + plasminogen. Afterward, LPL activity was assessed following the addition of fluorescent lipase substrate and calculated as a percent of vehicle control. Results are shown as the mean ± SD (n = 8 from three independent experiments) (∗*P* < 0.001 vs. ANGPTL3/8 alone). C: Samples from Figure 1B that contained ANGPTL3/8 were assessed using ANGPTL3 antibody Western blotting. Results are representative of three independent experiments, with two replicates for each experiment (total n = 6 from three independent experiments). ANGPTL, angiopoietin-like protein; LPL, lipoprotein lipase; tPA, tissue plasminogen activator.

To examine the ability of ANGPTL3/8 to inhibit LPL under conditions in which ANGPTL4/8 facilitates tPA-mediated plasmin generation, we preincubated LPL stable–expressing cells in the absence or presence of ANGPTL4/8, followed by an incubation in the absence or presence of tPA + plasminogen. As Fig. 1A shows, ANGPTL4/8 alone inhibited LPL activity, and this inhibition was completely reversed by the addition of tPA + plasminogen, consistent with our previous observations (11). The same experiment was also performed except that prior to the addition of lipase substrate, the cells were incubated in the absence or presence of ANGPTL3/8 at its approximate IC_80_ concentration. As expected, ANGPTL3/8 alone potently inhibited LPL, however this inhibition was almost completely reversed when ANGPTL4/8 + tPA + plasminogen were added (Fig. 1B). In contrast, in the absence of ANGPTL4/8, the addition of tPA + plasminogen did not affect ANGPTL3/8-mediated LPL inhibition.

Upon observing these results, we hypothesized that a possible explanation was that ANGPTL4/8-mediated plasmin generation resulted in plasmin cleavage of not only the ANGPTL4/8 complex but also the fibrinogen-like domain–containing ANGPTL3/8 complex. To test this hypothesis, one part of each sample analyzed for LPL activity in Fig. 1B was also examined via ANGPTL3 Western blotting. Figure 1C shows the results from these experiments, in which ANGPTL3 present in the ANGPTL3/8 complex was nearly completely degraded when ANGPTL4/8 + tPA + plasminogen were present, suggesting that plasmin-mediated ANGPTL3/8 cleavage could be the mechanism responsible for the suppression of ANGPTL3/8-mediated LPL inhibition in adipose tissue after feeding.

### ANGPTL4/8-mediated plasmin generation reduces LPL inhibition by ANGPTL4 and ANGPTL3

**Fig. 2:**
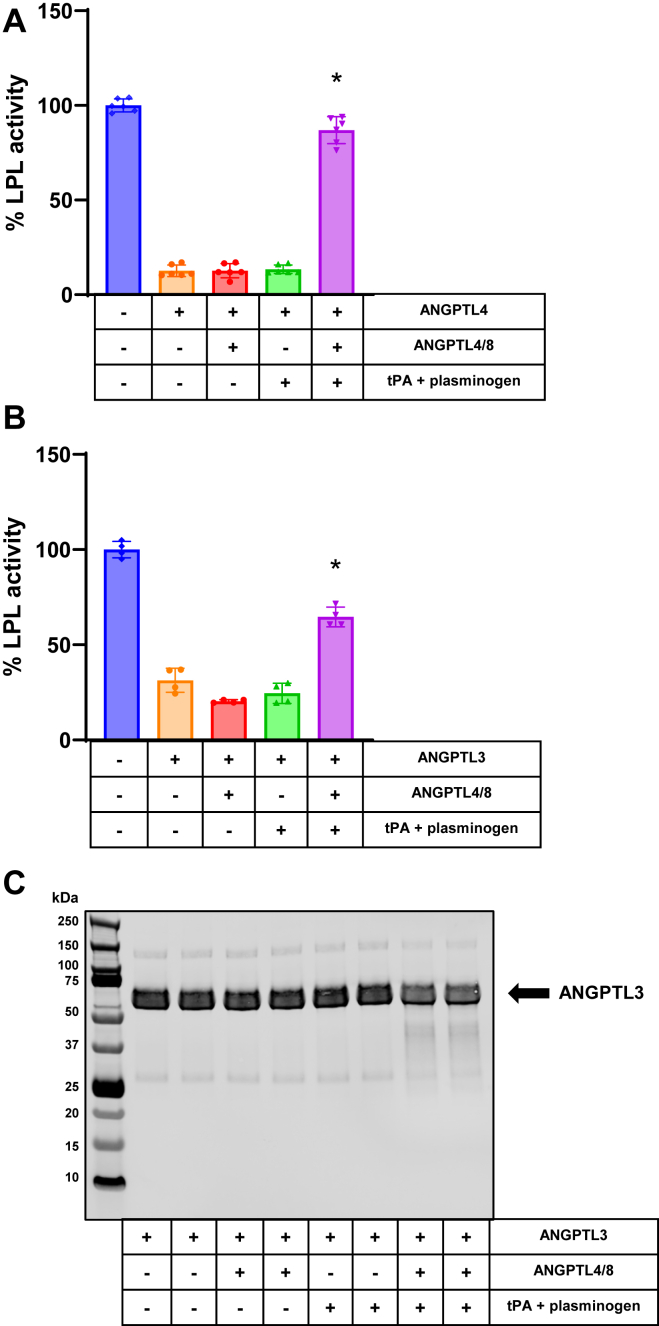
ANGPTL4/8-mediated plasmin generation blocks LPL inhibition by ANGPTL4 and ANGPTL3. A: LPL stable expression cells were incubated with vehicle alone throughout the experiment or in the absence or presence of ANGPTL4 after being previously preincubated with vehicle alone, ANGPTL4/8 alone, tPA + plasminogen, or ANGPTL4/8 + tPA + plasminogen. Afterward, LPL activity was assessed following the addition of fluorescent lipase substrate and calculated as a percent of vehicle control. Results are shown as the mean ± SD (n = 6 from two independent experiments) (∗*P* < 0.001 vs. ANGPTL4 alone). B: LPL stable expression cells were incubated with vehicle alone throughout the experiment or in the absence or presence of ANGPTL3 after being previously preincubated with vehicle alone, ANGPTL4/8 alone, tPA + plasminogen, or ANGPTL4/8 + tPA + plasminogen. Afterward, LPL activity was assessed following the addition of fluorescent lipase substrate and calculated as a percent of vehicle control. Results are shown as the mean ± SD (n = 4 from two independent experiments) (∗*P* < 0.001 vs. ANGPTL3 alone). C: Samples from Figure 2B that contained ANGPTL3 were assessed using ANGPTL3 Western blotting. Results are representative of two independent experiments with two replicates for each experiment (total n = 4 from two independent experiments). ANGPTL, angiopoietin-like protein; LPL, lipoprotein lipase; tPA, tissue plasminogen activator.

After contemplating these data, we considered the possibility that the LPL inhibitory effect of localized ANGPTL4 might also be blocked by ANGPTL4/8-mediated plasmin generation. To examine the ability of ANGPTL4 to inhibit LPL under conditions in which ANGPTL4/8 increases tPA-mediated plasmin generation, we preincubated the LPL stable–expressing cells in the absence or presence of ANGPTL4/8, followed by an incubation in the absence or presence of tPA + plasminogen. Afterward, we added ANGPTL4 at its approximate IC_90_ concentration prior to the addition of lipase substrate. As shown in Fig. 2A, ANGPTL4 strongly inhibited LPL activity, but this inhibition was dramatically overcome when ANGPTL4/8 + tPA + plasminogen were present. In contrast, in the absence of ANGPTL4/8, the addition of tPA + plasminogen was unable to reduce ANGPTL4-mediated LPL inhibition. Because ANGPTL4/8 and ANGPTL4 were both present in these experiments and because we have previously shown that ANGPTL4 in the ANGPTL4/8 complex is rapidly cleaved by tPA + plasminogen (11), ANGPTL4 Western blotting experiments to look for ANGPTL4 cleavage could not be performed.

We next considered the circulating LPL inhibitor ANGPTL3 and whether its ability to inhibit LPL would also be blocked by ANGPTL4/8 + tPA + plasminogen. ANGPTL3 is a much less potent LPL inhibitor than ANGPTL3/8, but it circulates at much higher concentrations (9). To examine the ability of ANGPTL3 to inhibit LPL under conditions in which ANGPTL4/8 facilitates tPA-mediated plasmin generation, we preincubated the LPL-expressing cells in the absence or presence of ANGPTL4/8, followed by an incubation in the absence or presence of tPA + plasminogen. Afterward, we added ANGPTL3 at its approximate IC_70_ concentration before the addition of lipase substrate.

As shown in Fig. 2B, ANGPTL3 inhibited LPL activity, and this inhibition was markedly but not fully reversed when ANGPTL4/8 + tPA + plasminogen were present. In the absence of ANGPTL4/8, addition of tPA + plasminogen did not reduce ANGPTL3-mediated LPL inhibition. To determine if ANGPTL4/8-mediated plasmin generation could also cleave ANGPTL3 alone, we performed ANGPTL3 Western blotting on one part of each sample shown in Fig. 2B. As Fig. 2C demonstrates, ANGPTL3 appeared to be partially degraded when ANGPTL4/8 +tPA + plasminogen were present. These data suggested that plasmin-mediated ANGPTL3 modification could also be responsible for the suppression of ANGPTL3-mediated LPL inhibition.

As Fig. 2C shows, there was a faster migrating band that was also present Fig. 1C at a somewhat greater intensity. The fact that this band was present in both instances suggested that it might be a minor ANGPTL3 degradation product, although this could not be concluded with certainty. Because ANGPTL3/8 was a more potent LPL inhibitor than ANGPTL3, the respective activity assays corresponding to the Western blots were conducted with less ANGPTL3/8 than ANGPTL3. As a result, the ratio of albumin to ANGPTL3/8 was higher than the ratio of albumin to ANGPTL3. This likely explains why the ANGPTL3 band in Fig. 1C appears to be somewhat compressed by albumin, while the ANGPTL3 band from Fig. 2C does not. This compression did not meaningfully affect the ability to show that ANGPTL3 in the ANGPTL3/8 complex was almost completely degraded in the presence of ANGPTL4/8 + tPA + plasminogen. In contrast, the average intensity of the ANGPTL3 bands in the presence of ANGPTL4/8 + tPA + plasminogen in Fig. 2C decreased by 26.2% compared to control, consistent with the marked, but not complete, restoration of LPL activity observed in the corresponding enzymatic assay.

### ANGPTL4/8-mediated plasmin generation prevents ApoC3 from inhibiting LPL

**Fig. 3:**
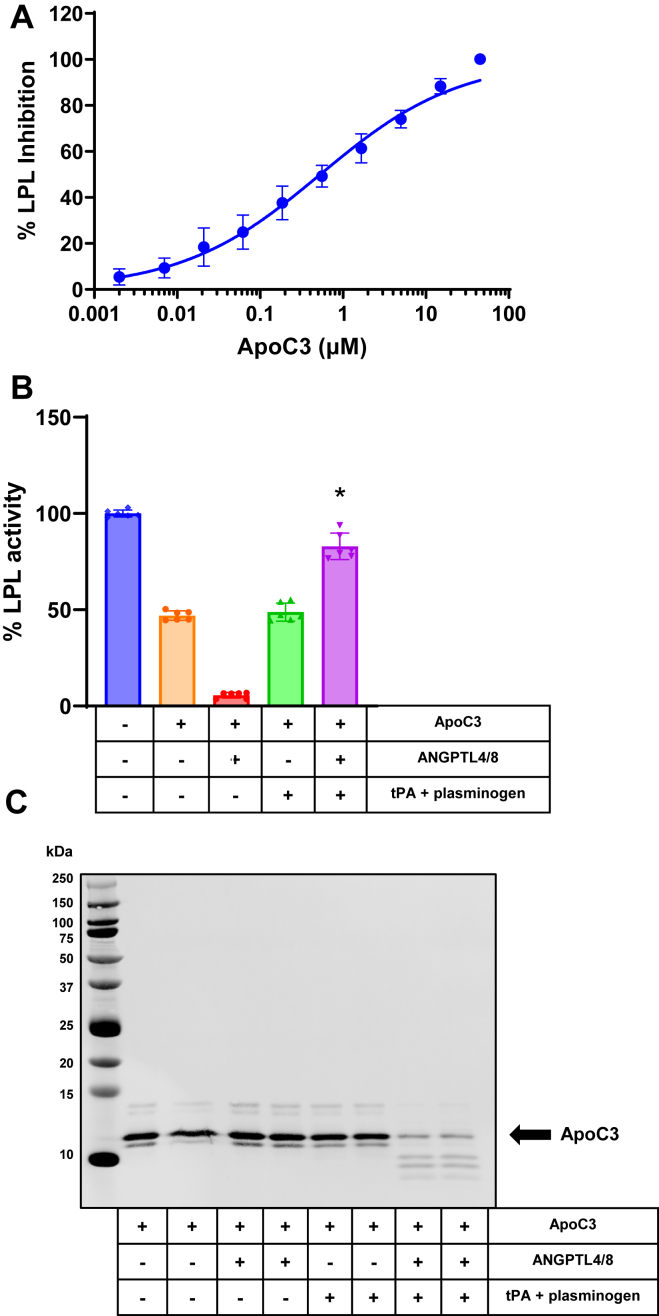
ANGPTL4/8-mediated plasmin generation blocks ApoC3-mediated LPL inhibition. A: The effect of increasing concentration of ApoC3 on LPL enzymatic activity was assessed using LPL stable–expressing cells and fluorescent lipase substrate. Results are shown as the mean ± SD (n = 6 from three independent experiments). B: LPL stable expression cells were incubated with vehicle alone throughout the experiment or in the absence or presence of ApoC3 after being previously preincubated with vehicle alone, ANGPTL4/8 alone, tPA + plasminogen, or ANGPTL4/8 + tPA + plasminogen. Afterward, LPL activity was assessed following the addition of fluorescent lipase substrate and calculated as a percent of vehicle control. Results are shown as the mean ± SD (n = 6 from two independent experiments) (∗*P* < 0.001 vs. ApoC3 alone). C: Samples from Figure 3B that contained ApoC3 were assessed using ApoC3 Western blotting. Results are representative of two independent experiments with two replicates for each experiment (total n = 4 from two independent experiments). ANGPTL, angiopoietin-like protein; LPL, lipoprotein lipase; tPA, tissue plasminogen activator.

We next considered what the possible effect of ANGPTL4/8-mediated plasmin generation might be on ApoC3-mediated LPL inhibition. ApoC3 is a relatively weak inhibitor of LPL but circulates at extremely high concentrations (14, 15, 16). To assess this, we first determined the amount of ApoC3 to use by examining the effects of increasing concentrations of ApoC3 on LPL activity by using LPL-expressing cells and fluorescent lipase substrate. As Fig. 3A shows, ApoC3 dose dependently inhibited LPL activity, albeit in a much less potent manner than ANGPTL3/8, ANGPTL3, or ANGPTL4, with an approximate IC_50-60_ of 0.5 μM. We then used this concentration of ApoC3 to test the effects of ANGPTL4/8-mediated plasmin generation on ApoC3-mediated LPL inhibition.

In these experiments, we again preincubated the LPL stable–expressing cells in the absence or presence of ANGPTL4/8, followed by an incubation in the absence or presence of tPA + plasminogen. Afterward, we incubated the cells with ApoC3 at its approximate IC_50-60_ concentration before adding lipase substrate. As shown in Fig. 3B, ApoC3 alone inhibited LPL activity, and the combination of ApoC3 + ANGPTL4/8 almost completely nullified LPL activity, suggesting that the two proteins may have had very different mechanisms of LPL inhibition that were additive with each other. Remarkably, this inhibition was largely, but not fully, decreased when tPA + plasminogen were added to the combination of ApoC3 + ANGPTL4/8. Without ANGPTL4/8 present, however, addition of tPA + plasminogen did not affect ApoC3-mediated LPL inhibition. To see if ANGPTL4/8-mediated plasmin generation could cleave ApoC3, we performed ApoC3 Western blotting on one part of each sample shown in Fig. 3B. As Fig. 3C demonstrates, ApoC3 was mostly, but not completely, degraded when ANGPTL4/8 + tPA + plasminogen were present, suggesting that plasmin-mediated ApoC3 cleavage might be the mechanism responsible for the decreased ability of ApoC3 to inhibit LPL activity.

### ApoC2-mediated LPL stimulation is unaffected by ANGPTL4/8-mediated plasmin generation

**Fig. 4:**
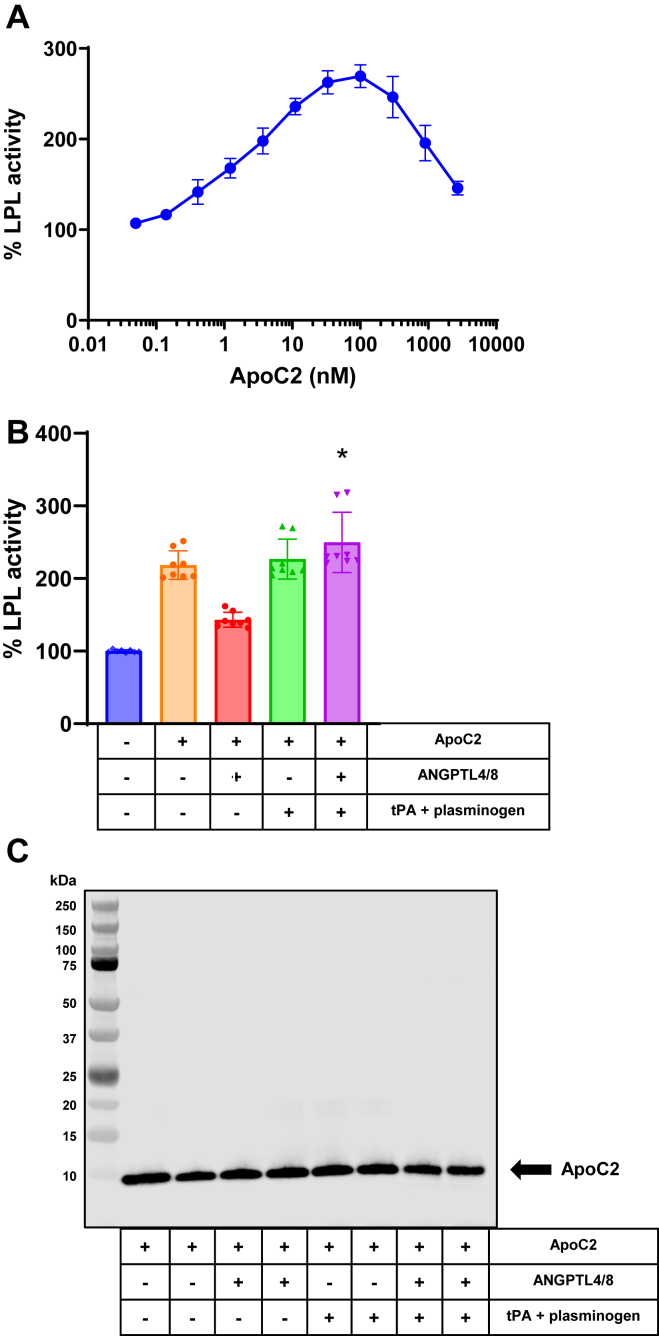
ANGPTL4/8-mediated plasmin generation does not block ApoC2-mediated LPL stimulation. A: The effect of increasing concentrations of ApoC2 on LPL enzymatic activity were assessed using LPL stable–expressing cells and fluorescent lipase substrate. Results are shown as the mean ± SD (n = 6 from three independent experiments). B: LPL stable expression cells were incubated with vehicle alone throughout the experiment or in the absence or presence of ApoC2 after being previously preincubated with vehicle alone, ANGPTL4/8 alone, tPA + plasminogen, or ANGPTL4/8 + tPA + plasminogen. Afterward, LPL activity was assessed following the addition of fluorescent lipase substrate and calculated as a percent of vehicle control. Results are shown as the mean ± SD (n = 8 from three independent experiments) (∗*P* < 0.001 vs. ApoC2 + ANGPTL4/8). C: Samples from Figure 4B that contained ApoC2 were assessed using ApoC2 Western blotting. Results are representative of three independent experiments with two replicates for each experiment (total n = 6 from three independent experiments). ANGPTL, angiopoietin-like protein; LPL, lipoprotein lipase; tPA, tissue plasminogen activator.

Finally, we considered the fact that ApoC2 is a critical activator of LPL activity as shown by the fact that ApoC2 deficiency results in severe hypertriglyceridemia (23, 24, 25, 26, 27) and wondered what the effects of ANGPTL4/8-mediated plasmin generation might be on ApoC2-mediated stimulation of LPL activity. To assess this, we first examined the effects of increasing concentrations of ApoC2 on LPL activity by using LPL stable–expressing cells and fluorescent lipase substrate. As Fig. 4A shows, ApoC2 stimulated LPL activity in a bell-shaped manner, consistent with our previous results observed for the effect of ApoC2 on glycosylphosphatidylinositol high-density lipoprotein–binding protein 1–LPL (27). Based on these results, we chose an ApoC2 concentration of 50 nM for subsequent experiments to test the effects of ANGPTL4/8-mediated plasmin generation.

In these experiments, we preincubated the LPL stable–expressing cells in the absence or presence of ANGPTL4/8, followed by an incubation in the absence or presence of tPA + plasminogen. Afterward, we incubated the cells in the presence of ApoC2 prior to adding lipase substrate. As shown in Fig. 4B, ApoC2 markedly stimulated LPL activity and ANGPTL4/8-mediated plasmin generation did not decrease the ability of ApoC2 to increase LPL activity. Based on these results, we did not expect that ANGPTL4/8-mediated plasmin generation would have caused any cleavage of ApoC2 to occur. To determine if this was the case, we performed ApoC2 Western blotting on one half of each sample shown in Fig. 4B. As shown in Fig. 4C, ApoC2 remained intact in the presence of ANGPTL4/8 + tPA + plasminogen, consistent with the observation that ANGPTL4/8-mediated plasmin generation did not affect ApoC2 functionality.

### Characterization of ANGPTL4/8-mediated plasmin generation and its effects on LPL

**Fig. 5:**
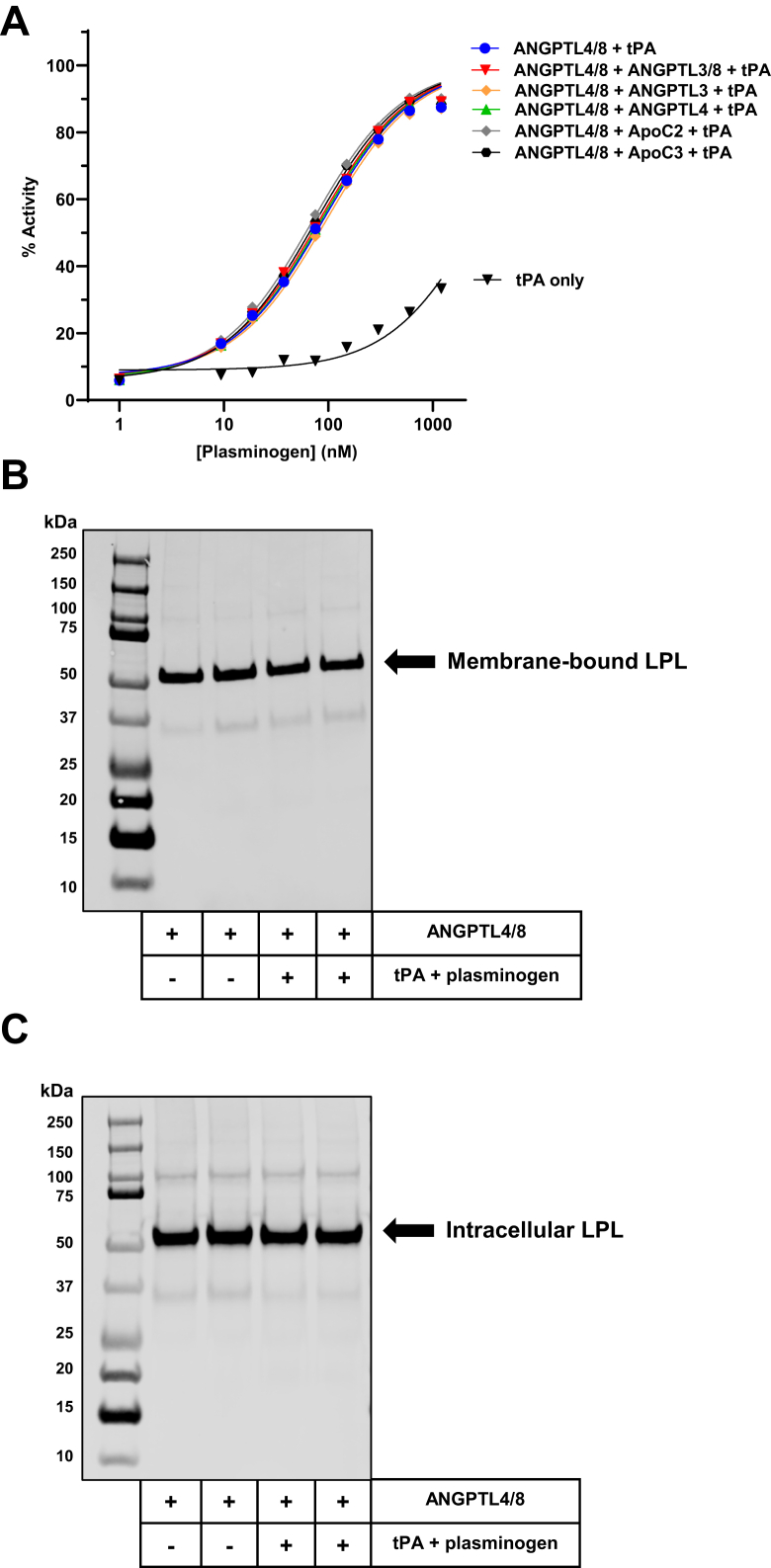
Characterization of ANGPTL4/8-mediated plasmin generation and its effects on LPL. A: The ability of tPA to convert plasminogen to plasmin in the presence of ANGPTL4/8 was examined after addition of ANGPTL3/8, ANGPTL3, ANGPTL4, ApoC3, or ApoC2, with the molar ratio of each protein to ANGPTL4/8 being the same as in the LPL activity assays. Generation of plasmin was measured using colorimetric plasmin activity assays, with absorbances recorded spectrophotometrically. Results shown are representative of three independent experiments. B: LPL stable–expressing cells were preincubated with ANGPTL4/8, followed by incubation in the absence or presence of tPA + plasminogen. Cells were washed, and heparin was added to release membrane-bound LPL, which was measured via Western blotting. Results are presentative of 6 replicates from 2 independent experiments. C: Following removal of membrane-bound LPL, cells were solubilized using RIPA buffer, and LPL present within the cells was analyzed via Western blotting. Results are presentative of six replicates from two independent experiments. ANGPTL, angiopoietin-like protein; LPL, lipoprotein lipase; tPA, tissue plasminogen activator.

After observing the results in Fig. 1, Fig. 2, Fig. 3, Fig. 4, we considered if the addition of any of the proteins tested might decrease ANGPTL4/8-mediated plasmin generation. If this were to happen, it might explain the incomplete reversal of ANGPTL3-mediated and ApoC3-mediated LPL inhibition. To test this idea, we examined the ability of ANGPTL4/8 to generate plasmin in the presence of tPA + plasminogen both alone and when each of the additional proteins tested were added at the exact same molar ratios to ANGPTL4/8 that were studied in the LPL activity assays, Fig. 5A shows the results from these experiments in which plasmin generation was measured using plasmin activity assays. Consistent with our previous observations, in the presence of tPA and plasminogen only, minimal plasmin activity was generated, while the addition of ANGPTL4/8 markedly increased the amount of plasmin generation (11). Interestingly, ANGPTL4/8-mediated plasmin generation was largely unaffected by any of the proteins tested, suggesting that reduction of ANGPTL4/8-mediated plasmin generation was likely not the mechanism responsible for the incomplete reversal of ANGPTL3-mediated or ApoC3-mediated LPL inhibition.

In a previous study, we demonstrated that during ANGPTL4/8-mediated plasmin generation and degradation of the ANGPTL4/8 complex, there was no increase in the amount of LPL released into the media (11). To address whether ANGPTL4/8-mediated plasmin generation modified the amount of LPL present on the cell surfaces, we incubated ANGPTL4/8 pretreated LPL stable–expressing cells in the absence or presence of tPA + plasminogen. Afterward, the cells were washed, and any LPL bound to the cell membranes was released by adding heparin and measured via Western blotting with an anti-LPL antibody. Figure 5B shows the results from these experiments in which there was no appreciable change in the amount of LPL on the cell surfaces when tPA and plasminogen were added to ANGPTL4/8. At the same time, LPL present within the cells was also measured by Western blotting with the same anti-LPL antibody. As shown in Fig. 5C, the amount of LPL present within the cells also remained relatively constant after the addition of tPA and plasminogen to ANGPTL4/8-treated cells. Together, the results from these experiments suggested that the regulation of LPL activity following ANGPTL4/8-mediated plasmin generation did not affect the levels of plasma membrane–bound LPL or intracellular LPL.

## Discussion

In a previous study, we demonstrated that the ANGPTL4/8 complex binds tPA + plasminogen to generate plasmin, which then cleaves ANGPTL4/8 to restore LPL activity, and that this effect could be mimicked by addition of plasmin (11). The major observation in the current study is that plasmin generated by the ANGPTL4/8 complex in the presence of tPA + plasminogen not only cleaves the ANGPTL4/8 complex itself to restore LPL activity but also cleaves the fibrinogen-like domain–containing ANGPTL3/8 complex to protect LPL from ANGPTL3/8-mediated LPL inhibition. In addition, ANGPTL4/8-mediated plasmin generation also largely blocked the ability of ANGPTL4 to inhibit LPL activity and markedly (but not completely) blocked the ability of ANGPTL3 and ApoC3 to inhibit LPL activity.

Reduction of ANGPTL4/8-mediated plasmin generation by ANGPTL3 or ApoC3 did not appear to be the mechanism responsible for the incomplete reversal of ANGPTL3-mediated or ApoC3-mediated LPL inhibition, suggesting that further investigation will be needed to understand the reasons for the incomplete reversal of LPL inhibition by ANGPTL3 and ApoC3. In addition, our experiments measuring LPL on cell surfaces and within the cells indicated that ANGPTL4/8-mediated plasmin generation did not affect the amount of LPL on the cell membranes, suggesting that regulation of LPL activity by the ANGPTL4/8 complex may occur on the surfaces of adipose tissue endothelial cells in vivo.

Importantly, the ability of the crucial LPL activator ApoC2 to stimulate LPL activity was unaffected by ANGPTL4/8-mediated plasmin generation. Together, these results therefore answer a major unresolved question from our previous study (11), which was if ANGPTL4/8 generates plasmin for self-cleavage to remove ANGPTL4/8 from LPL and restore maximal LPL activity, how can LPL be protected from inhibition by ANGPTL3/8, the most potent circulating LPL inhibitor, as well as other circulating LPL inhibitors such as ANGPTL3 and ApoC3, and the localized LPL inhibitor ANGPTL4.

**Fig. 6:**
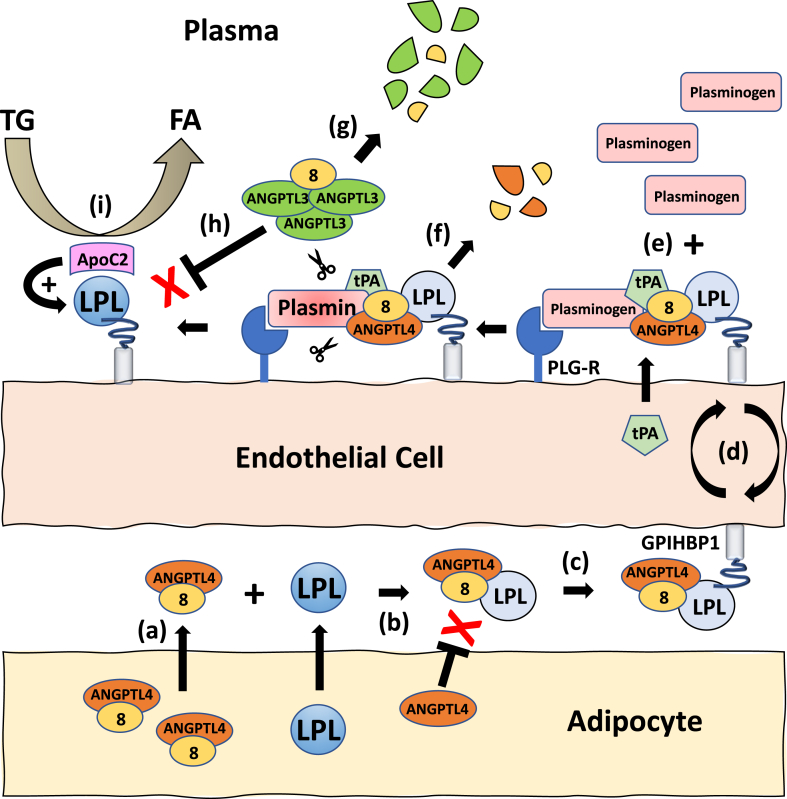
An updated model for adipose tissue LPL regulation in the postprandial state. After feeding, increased ANGPTL4/8 (a) binds LPL, protects it from inactivation by ANGPTL4, but also partially inhibits LPL (light blue color) (b). Partially active ANGPTL4/8-LPL binds GPIHBP1 on abluminal endothelial surfaces (c) and is translocated (d). Concurrently, tPA secreted by the endothelium and plasminogen present on endothelial plasminogen receptors (PLG-R) bind the LPL-ANGPTL4/8 complex on luminal capillary surfaces (e). ANGPTL4/8 increases tPA-mediated generation of plasmin, which cleaves ANGPTL4/8 (f) to restore LPL activity (dark blue color). The plasmin generated (g) also protects LPL from inhibition by circulating ANGPTL3/8, ANGPTL3, and ApoC3 as well as from any localized ANGPTL4 that may be present (for the purpose of clarity only ANGPTL3/8 is shown in the figure) (h), while permitting ApoC2-mediated LPL stimulation to occur (i). ANGPTL, angiopoietin-like protein; LPL, lipoprotein lipase; tPA, tissue plasminogen activator.

Figure 6 shows an updated model for how we now believe LPL activity is regulated in adipose tissue postprandially. After feeding, ANGPTL8 increases, and therefore local ANGPTL4/8 levels increase (a). The ANGPTL4/8 complex acts like a bodyguard for LPL (b) as it transits from the subendothelial space (c) to capillary luminal surfaces (d). Along the way, the ANGPTL4/8–LPL complex binds tPA secreted by endothelial cells and plasminogen that is localized on plasminogen receptors present on the luminal endothelial surface of adipose tissue capillaries (e) (28). ANGPTL4/8 then facilitates tPA-mediated conversion of plasminogen to plasmin, which cleaves the LPL-bound ANGPTL4/8 to remove ANGPTL4/8 from LPL, resulting in restoration of LPL activity (f). The plasmin generated also cleaves fibrinogen-like domain–containing ANGPTL3/8 in the vicinity (g) to protect LPL from inhibition by increased postprandial concentrations of ANGPTL3/8 (h). In addition, the plasmin generated suppresses the ability of the circulating LPL inhibitors ANGPTL3 and ApoC3 to inhibit LPL and is also able to block LPL inhibition caused by any localized ANGPTL4 that may be present (for simplicity, the figure only shows ANGPTL3/8 cleavage). Interestingly, the generation of plasmin does not affect the ability of ApoC2 to activate LPL, thus allowing ApoC2 to fully stimulate LPL activity so that LPL can be maximally active in adipose tissue capillaries after feeding (i).

In our current study, the addition of tPA + plasminogen to ANGPTL4/8 was essential to protect LPL from inhibition by ANGPTL3/8 and ANGPTL4. In a previous study several years ago, we showed that ANGPTL4/8 could protect LPL from inhibition by ANGPTL3/8 and ANGPTL4 without tPA and plasminogen being added (9) Those experiments, however, were performed in media containing 10% FBS, which provided a source of plasminogen and tPA (9). After discovering that ANGPTL4/8 was capable of binding plasminogen and tPA (11), we replaced FBS with 0.1% BSA to allow for precise control of the addition of tPA and plasminogen to the media. Under these conditions, the presence of tPA and plasminogen was clearly required for the ability of the ANGPTL4/8 complex to protect LPL from inhibition by ANGPTL3/8 and ANGPTL4.

A limitation of our study is that it consists mainly of in vitro biochemical findings and that much additional investigation will be required to ascertain the relevance of our findings to TG metabolism in vivo. Despite this limitation, however, our data convincingly demonstrate a plausible mechanism by which the ANGPTL4/8 complex ensures that adipose tissue LPL can be fully active postprandially. This occurs through ANGPTL4/8-mediated generation of plasmin, which blocks LPL inhibition by the key LPL-inhibitory proteins and complexes (ANGPTL3/8, ANGPTL3, ANGPTL4, and ApoC3) without interfering with LPL activation by ApoC2.

A somewhat surprising finding from our study was that ANGPTL4/8-mediated plasmin generation resulted in suppression of ApoC3-mediated LPL inhibition, while having no effect on ApoC2-mediated stimulation of LPL. These data were consistent with the subsequent observation that the addition of ANGPTL4/8 + tPA + plasminogen resulted in cleavage of ApoC3 but not ApoC2. While plasmin-mediated cleavage of ANGPTL3 as part of the ANGPTL3/8 complex is consistent with the concept that ANGPTL3 contains a fibrinogen-like domain and that fibrin is a natural substrate for plasmin (14, 15, 16, 17, 18, 19, 20), there is no obvious fibrinogen-like domain present in ApoC3. A clear further area of investigation will be to focus on trying to map the exact plasmin cleave site (or sites) residing in ApoC3.

Our findings build upon those of many other researchers who have studied the ANGPTL3/4/8 protein family, including Zhang and colleagues, who provided some of the earliest insights into ANGPTL8 biology, proposed an elegant model for how this family of proteins works to control TG metabolism in the fed versus fasted state and then further refined their model (29, 30, 31, 32, 33, 34, 35). In addition, the Kersten, Ploug, and Young laboratories each performed pioneering work on ANGPTL4, including elucidating the biochemical mechanism by which ANGPTL4 potently inhibits LPL, leading to an understanding of how and why ANGPTL4 functions to inhibit LPL enzymatic activity in adipose tissue (6, 7, 8, 36, 37, 38, 39, 40, 41).

In addition, the research performed by Hobbs and coworkers was critical in the discovery and identification of ANGPTL8, its caloric responsiveness at the RNA level, its interaction with ANGPTL3, and the development of whole body and tissue-specific *Angptl8* knockout mice (13, 19, 20, 42, 43, 44, 45, 46, 47). In a seminal paper, this group demonstrated that while liver-specific *Angptl8* knockout (which eliminates the liver-derived circulating ANGPTL3/8 complex) was beneficial and resulted in markedly reduced TG levels, adipose-specific *Angptl8* knockout (which eliminates adipose tissue–derived, localized ANGPTL4/8 complex) caused significantly increased postprandial circulating TG concentrations (47). In retrospect, this report provided the most important in vivo data demonstrating that ANGPTL8 truly had two diametrically opposed aspects of its function, with the first being to work via the circulating ANGPTL3/8 complex in an endocrine manner to potently inhibit LPL activity in oxidative tissues and the second being to work via localized formation of ANGPTL4/8 complexes in adipose tissue in an autocrine/paracrine manner to preserve LPL activity. Our own current study thus begins to provide some of the mechanistic details by which ANGPTL4/8 works in the fat to ensure that LPL is both fully protected and maximally active in the postprandial state so that TG can be efficiently stored in the adipose tissue and not deposited ectopically in other tissues such as the liver.

Over the past several years, we have often wondered how the mechanisms of all the different critical proteins that affect LPL activity might be unified to provide a holistic model for the control of TG metabolism. This has been a daunting task due to the seemingly very disparate circulating levels of these proteins, their localized versus systemic effects, the divergent effects they have on LPL activity, and their vastly different potencies. A major goal of our own laboratory has been to reconcile how the ANGPTL3/4/8 family members can work together not only with each other but also with the key apolipoproteins ApoC2, ApoC3, and ApoA5 to control TG metabolism though selective modulation of LPL enzymatic activity in a tissue-specific manner. Our recent discovery that ApoA5 acts by selectively suppressing the ability of the ANGPTL3/8 complex to inhibit LPL provided the first direct linkage between ANGPTL3/4/8 family members and apolipoproteins (48, 49). However, this work left unanswered what the possible connections between ANGPTL3/4/8 protein family members might be with ApoC3 and ApoC2 (50). Our current study has begun to fill in some of these gaps by showing that the ANGPTL4/8 complex can act to block the LPL-inhibitory activity of ApoC3, while allowing full ApoC2-mediated stimulation of LPL activity to occur.

While far from providing a complete explanation for how LPL activity is modulated to control TG metabolism, our observations have begun to fill in some of the missing puzzle pieces and have established unique connections between the major apolipoprotein players in TG metabolism (ApoC2, ApoC3, and ApoA5) and the ANGPTL3/4/8 protein family. Clearly, much additional investigation will be required to understand in greater depth the exact mechanisms mediating each of these various interactions as well as their functional relevance to TG metabolism in vivo.

## Data availability

All data are included in the main text and figures.

## Conflict of interest

The authors declare no conflicts of interest with the contents of this article.
